# Nitrite as Direct S-Nitrosylating Agent of Kir2.1 Channels

**DOI:** 10.1155/2014/517126

**Published:** 2014-07-16

**Authors:** Gabriella Montesanti, Maria Laura Parisella, Giusi Garofalo, Daniela Pellegrino

**Affiliations:** Department of Biology, Ecology and Earth Sciences (DiBEST), University of Calabria, Arcavacata di Rende, 87036 Cosenza, Italy

## Abstract

Nitrite, a physiological nitric oxide (NO) storage form and an alternative way for NO generation, affects numerous biological processes through NO-dependent and independent pathways, including the S-nitrosylation of thiol-containing proteins. Mechanisms underlying these phenomena are not fully understood. The purpose of this study was to analyse in the rat heart (as prototype of mammalian heart) whether nitrite affects S-nitrosylation of cardiac proteins and the potential targets for S-nitrosylation. Rat hearts, perfused according to Langendorff, were exposed to nitrite. By Biotin Switch Method, we showed that nitrite treatment increased the degree of S-nitrosylation of a broad range of membrane proteins. Further analysis, conducted on subfractioned proteins, allowed us to identify a high level of nitrosylation in a small range of plasmalemmal proteins characterized by using an anti-Kir2.1 rabbit polyclonal antibody. We also verified that this effect of nitrite is preserved in the presence of the NO scavenger PTIO (2-phenyl-4,4,5,5-tetramethylimidazoline-1-oxyl 3-oxide). Our results suggest, for the first time, that nitrite represents a direct S-nitrosylating agent in cardiac tissues and that inward-rectifier potassium ion channels (Kir2.1) are one of the targets. These observations are of relevance since they support the growing evidence that nitrite is not only a NO reserve but also a direct modulator of important functional cardiac proteins.

## 1. Introduction

The research on nitrite biology has exploded in recent years with special attention to the therapeutic potential of nitrite in cardiovascular diseases [1, 2]. It is widely accepted that nitrite homeostasis has an impact on cardiovascular health and may serve as an important predictor of cardiovascular risk [3]. Nitrite is a physiological, relevant storage form of nitric oxide (NO) in blood and tissues and represents an important alternative way for NO generation [4]. Our previous studies on the rat heart suggested that, under basal conditions, nitrite represents a nitric oxide synthase- (NOS-) independent source of NO, which modulates cardiac contractility through the classic cGMP/PKG pathway [5]. In addition, several studies showed that, in a variety of mammalian tissues, inorganic nitrite affects numerous biological processes also through NO-independent pathways including S-nitrosylation of thiol-containing proteins, normally associated to NO [6].

S-nitrosylation, the covalent attachment of a nitrogen monoxide group to the thiol side chain of cysteine, has emerged as an important mechanism for dynamic, posttranslational regulation of most or all main classes of protein, comparable to phosphorylation [7]. Numerous proteins have been characterized as targets for S-nitrosylation [3, 8, 9]. Alterations of protein S-nitrosylation in the heart tissue has been implicated in several pathological processes [2, 3, 10].

It is well established that bioactive products of NOS, principally NO and low-molecular-weight-S-nitrosothiols (SNOs), regulate a large array of signal transduction pathways, acting mainly through the S-nitrosylation of cysteine residues located at active or allosteric protein sites [7]. For example, several studies have highlighted the nitrosylation mechanisms at mitochondrial level underlying the cytoprotective effects exerted by both nitrite and NO [11, 12]. Important substrates of S-nitrosylation that influence cardiac function include receptors, enzymes, ion channels, transcription factors, and structural proteins. Cardiac ion channels subserving excitation-contraction coupling are regulated by S-nitrosylation [13, 14]. The shape and duration of the cardiac action potential are regulated by multiple ion channels (RyR2, SERCA2a, L-type calcium channel) that are subject to regulatory S-nitrosylation [13, 15]. In particular, the inward-rectifying K current (IK1) that plays a critical role in modulating cardiac excitability is highly sensitive to this type of control. As shown by Gómez and coworkers [16], S-nitrosylation of a single cysteine in the Kir2.1 channel protein is able to shorten action potential.

Protein S-nitrosylation is recognized to mediate NO signalling. However, it is largely unknown whether nitrite also contributes to S-nitrosylation. Few data show nitrite as S-nitrosylating agent, an effect which is ascribed to its conversion to NO, although NO production from nitrite occurs through several highly nitrosants intermediates [3].

In light of these findings, the purpose of the present study was to examine, on the rat heart, the direct S-nitrosylating effect of nitrite and to identify potential cardiac targets for protein S-nitrosylation.

## 2. Materials and Methods

### 2.1. Animals

Male Wistar rats (Morini, Bologna, Italy), weighing 180–250 g, were housed three per cage in a ventilated cage rack system and were fed ad libitum. All studies were performed in accordance with the Guide for the Care and Use of Laboratory Animals published by the US National Institute of Health (publication no. 85-23, revised 1996).

### 2.2. Langendorff Preparation

Rats were anaesthetized with ethyl carbamate (2 g/Kg rat, i.p.) and the hearts rapidly excised and transferred in ice-cold buffered Krebs-Henseleit solution (KHs). The aorta was immediately cannulated with a glass cannula and connected with the Langendorff apparatus to start perfusion at a constant flow-rate of 12 mL/min as previously described [17]. Hemodynamic parameters were assessed using a PowerLab data acquisition system and analysed using chart software (AD Instruments, Basile, Italy). Exposure to pharmacological agents (sodium nitrite, GSNO, NONOATE, PTIO) was obtained by perfusing the cardiac preparations with KHs containing the specific substance at the desired concentration. After 20 minutes perfusion was stopped and the hearts were rapidly immersed in liquid nitrogen to be stored at −80°C.

### 2.3. Biotin Switch

The biotin switch assay was performed essentially as previously described [18, 19]. In this method, unmodified protein thiols are blocked, while S-nitrosylated thiols are reduced to free thiols; the newly generated thiols are labeled with a biotin tag, followed by avidin capture of the labeled proteins. The final products then correspond to the originally S-nitrosylated proteins that can be identified by proteomic approaches. Ventricles were homogenized on ice in 20 mM Tris pH 7.5, 150 mM NaCl, 1% Igepal CA 630, 0.5% sodium deoxycholate, 1 mM EDTA, 0.1% SDS, 200 mM sodium orthovanadate, and protease inhibitor cocktail, using a polytron tissue grinder. The homogenate was centrifuged at 4°C for 60 min at 100.000 g. The supernatant, containing cytosolic proteins, was collected and the pellet, containing membrane proteins, was resuspended in homogenization buffer or subfractioned by linear concentration gradient of sucrose; proteins were quantified with Bradford reagent and cytosolic contamination was excluded by using anti-GADPH policlonal antibody (negative control; data not shown). Extracts were adjusted to 0.5 mg/mL of protein and equal amounts were blocked with 4 volumes of blocking buffer (225 mM Hepes, pH 7.7, 0.9 mM EDTA, 0.09 mM neocuproine, 2.5% SDS, and 20 mM MMTS) at 50°C for 20 min with agitation. After blocking, extracts were precipitated with 2 volumes of cold (−20°C) acetone, chilled at −20°C for 10 min, centrifuged at 2000 g, 4°C for 5 min, washed with acetone, dried out at room temperature, and resuspended in 0.1 mL HENS buffer (250 mM Hepes, pH 7.7, 1 mM EDTA, 0.1 mM neocuproine, and 1% SDS) × mg of protein. Until this step, all operations were carried out in the dark. A 1/3 vol of biotin-HPDP 4 mM in DMF and ascorbate 1 mM were added and incubated for 1 h at room temperature. Proteins were acetone-precipitated again and resuspended in the same volume of HENS buffer. To detect biotinylated proteins by western blot, samples from the biotin switch assay were separated on 12% or 15% SDS-PAGE gels, transferred to PVDF membranes, blocked with nonfat dried milk, and incubated with streptavidin-peroxidase diluted 1/5000 for 1 h. Blots were developed by enhanced chemiluminescence (ECL) and placed in a film cassette with photograph film. Films were exposed for 30 s, developed, and fixed. In additional experiments, the membrane used for S-nitrosylation detection was stripped and reprobed using a rabbit polyclonal antibody anti-Kir2.1 (Santa Cruz Biotechnology; 1 : 1000). Immunoblots were digitalized and the densitometric analysis of the bands was carried out using WCIF Image J based on 256 grey values (0 = white; 256 = black). Quantification of the bands was obtained by measuring (5 times on each band) the mean optical density of a square area, after the background has been subtracted.

### 2.4. Chemicals

Biotin-HPDP was purchased from Pierce; ECL was purchased from Amersham; the other chemicals/drugs were purchased from Sigma unless otherwise indicated and prepared immediately before each experiment.

### 2.5. Statistics

The results of absorbance measurements and the grey values obtained from the densitometric analysis were expressed as means ± SE of 5 determinations for each sample. To test the difference between the groups, Student's *t*-test was performed. Statistical significance was established at ^*^ *P* < 0.01 and ^**^ *P* < 0.001.

## 3. Results

The biotin switch method, successfully used in various mammalian tissues and cell types, was utilized to assess whether exposure of the perfused rat heart to nitrite induces proteins S-nitrosylation. By this method we analysed, in both cytosolic and membrane cardiac extracts, the proteins containing S-nitrosylated cysteines showing that nitrite treatment increased the degree of S-nitrosylation of a broad range of plasmalemmal proteins.

**Figure 1:**
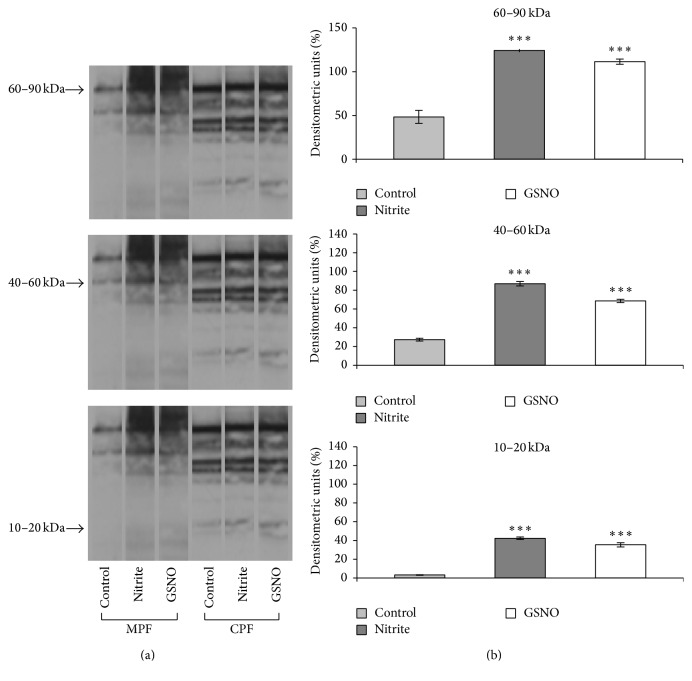
(a) Western blot of S-nitrosylated proteins in ventricle homogenates of control and nitrite or GSNO treated hearts: membrane (MPF) and cytosolic (CPF) protein fractions. Blot is representative of 3 independent experiments. (b) Amount of S-nitrosylation at the protein bands (MPF) that migrate at 60–90 kDa, 40–60 kDa, and 10–20 kDa. Data are means ± SE of 5 determinations for each animal (*n* = 3). Statistical differences were evaluated by Student's *t*-test; ^***^ *P* < 0.001.

Since a number of studies showed that nitrite mediates its effects through its conversion to NO, the changes in S-nitrosylation of nitrite-treated rat heart were compared with those induced by the NO donor GSNO. As shown in Figure 1(a), the analysis of S-nitrosylated proteins revealed that both nitrite and GSNO increased the degree of S-nitrosylation of a broad range of proteins which precipitate with the membrane fraction. In contrast, no differences in protein S-nitrosylation were observed in the cytosolic fraction. Nitrosylated membrane proteins are located in bands which migrate at 60–90 kDa, 40–60 kDa, and 10–20 kDa. Densitometric quantification of the blots (Figure 1(b)) revealed statistically significant increments in S-nitrosylation between control and nitrite-treated or GSNO-treated hearts.

**Figure 2:**
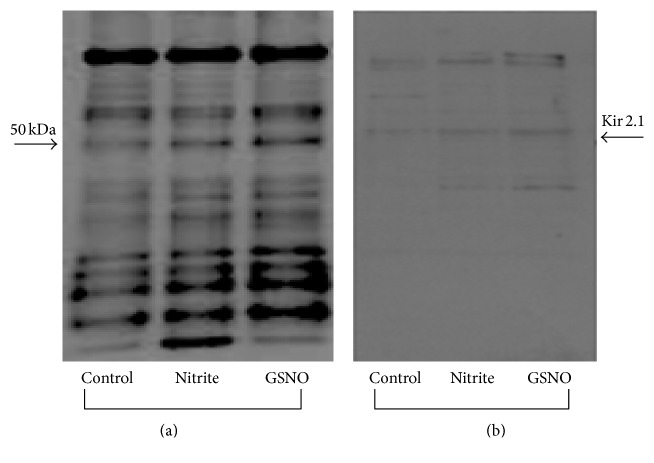
(a) Western blot of S-nitrosylated proteins in plasma membrane fraction of ventricle homogenates (isolated by linear concentration gradient of sucrose) of control and nitrite or GSNO treated hearts. Blot is representative of 3 independent experiments. (b) Membrane stripped and incubated with anti-Kir2.1 rabbit polyclonal antibody.

To further discriminate the proteins involved in the S-nitrosylation process, membrane fractions of control, nitrite, and GSNO-treated hearts were subfractioned by linear concentration gradient of sucrose. Results obtained in plasma membrane fraction showed that, after both nitrite and GSNO treatment, the more marked increase in S-nitrosylation is observable at a very specific location corresponding to proteins ranging from 45 to 50 kDa (Figure 2(a)). By immunodetection, the protein which undergoes S-nitrosylation in the presence of both nitrite and GSNO was identified as Kir2.1 channel (Figure 2(b)).

**Figure 3:**
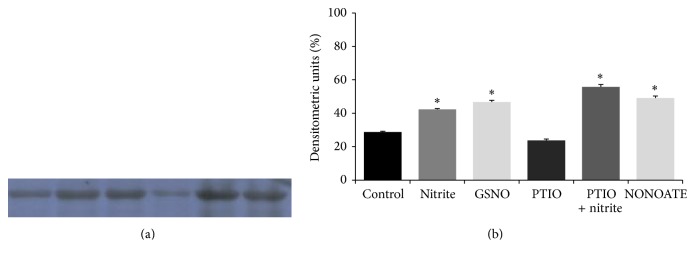
(a) Western blot of S-nitrosylated proteins in membrane fraction of ventricle homogenates of control, nitrite, GSNO, PTIO, PTIO + nitrite, and NONOATE treated heart. Blot is representative of 3 independent experiments. (b) Densitometric analysis of the protein band corresponding to 50 kDa. Data are means ± SE of 5 determinations for each animal (*n* = 3). Statistical differences were evaluated by Student's *t*-test; ^*^ *P* < 0.01.

To clarify if nitrite represents a direct S-nitrosylating agent for Kir2.1 or induces this effect through its conversion to NO, we evaluated the variations in S-nitrosylation in 45–50 kDa proteins after nitrite treatment alone and in the presence of the NO scavenger PTIO. We also assessed the nitrosylating effect of PTIO and two NO donors (GSNO and NONOATE). We found that the nitrite effect is preserved also in the presence of PTIO. PTIO alone did not change protein S-nitrosylation Figures 3(a) and 3(b).

## 4. Discussion

In the present report, to the best of our knowledge we demonstrate for the first time that nitrite represents a direct S-nitrosylating agent in cardiac tissues and Kir2.1 channels are one of its targets. These observations are of relevance since they support the growing evidence that nitrite is not only an NO reserve but also a direct modulator of important functional cardiac proteins.

This study represents a start point for understanding the potential direct mechanisms by which nitrite physiologically regulates heart function. Analysis of extracts from Langendorff perfused rat heart exposed to pharmacological doses of sodium nitrite shows an increase in the total level of cardiac SNO-proteins. It is well established that bioactive products of NO synthases, principally NO and SNOs, regulate a diverse array of signal transduction pathways, acting mainly through the covalent modification (S-nitrosylation) of cysteine residues located at active or allosteric sites of proteins [7]. As already reported [4, 20, 21], nitrite mediates its effects through its conversion to NO. Accordingly, we evaluated the effects of nitrite and GSNO, a biologically existing NO donor widely used as an* in vitro* S-nitrosylating agent [22]. By biotin switch method, we observed that both nitrite and GSNO increased the degree of S-nitrosylation, an effect which can be observed only at the level of membrane proteins.

In spite of the classic cardioprotective effects of nitrite (e.g., nitroglycerin has been used for long-term treatment of coronary heart disease or heart failure since the early 1800s, and it is still the drug of choice for the therapy of acute angina pectoris, [23]), only recently nitrite has been identified as an endocrine reservoir of NO that mediates physiological responses. Several studies showed that nitrite-dependent NO generation plays critical physiological and pathological roles and is controlled by oxygen tension, pH, reducing substrates, and nitrite levels. There are a variety of mechanisms of nitrite reduction to NO and it is now appreciated that this process, while being enhanced under hypoxic conditions, also occurs under normoxia [4, 20, 21]. In a previous study, we have shown that physiological concentrations of nitrite mediate a negative inotropic effect in the rat heart through a mechanism involving its reduction to NO and a following cGMP-PKG-dependent modulation of contractility [5]. However, accumulating evidence has suggested that nitrite may represent both a stable storage form of NO and a molecule with intrinsic signaling properties [24].

It is well established that S-nitrosylation is a key route for NO to directly modulate the function of many proteins [3, 7]. In the heart, protein S-nitrosylation has been implicated in several pathophysiological processes, such as ischemia, heart failure, and atrial fibrillation [22]. Several cardiac proteins are targets of S-nitrosylation. For example, the L-Type Ca^2+^ channel and the mitochondrial complex I were demonstrated to be S-nitrosylated and this appears to confer protective effects against ischemia/reperfusion injury [25, 26].

In order to detect nitrosylation targets, we used the biotin switch method, successfully adopted in various mammalian tissues and cell types (see [27] and references therein). We have subfractionated by linear concentration gradient of sucrose the S-nitrosylated membrane fractions of control, nitrite- and GSNO-treated hearts, showing a marked increase in S-nitrosylation after both nitrite and GSNO treatment in proteins migrating from 45 to 50 kDa. These proteins were subsequently identified by anti-Kir2.1 rabbit polyclonal antibody. Recently, Gómez et al. [16] reported that NO, under physiological conditions, increases cardiac IK1 by nitrosylation of cysteine 76 of Kir2.1 channels in both human atria and mouse ventricle. IK1 plays a key role in cardiac electrophysiology by stabilizing the resting membrane potential and shaping the initial and the final phase of the atrial and ventricular action potential [28]. Therefore, modulation of IK1 would have profound effects on cardiac excitability and arrhythmogenesis [29].

Since no significant qualitative and quantitative differences were observed in hearts exposed to nitrite and the NO donor GSNO, we speculated that the increase of S-nitrosylated proteins is mediated by NO and does not involve NO-independent mechanisms.

To address this point, we repeated experiments by treating the hearts with both NO donors (GSNO and NONOATE) and the NO scavenger PTIO. Our results demonstrated that the nitrite-induced increase in proteins S-nitrosylation at the location corresponding to Kir2.1 channels persists even in the presence of NO scavenging by PTIO. These results are relevant because they implicate that nitrite-induced S-nitrosylation is largely NO independent. As NO production from nitrite proceeds via HNO_2_ and N_2_O_3_ formation, which are both potent nitrosants [3], it remains to be clarified if one or both these intermediates are involved.

This study demonstrated that nitrite is a direct S-nitrosylating agent of many cardiac proteins, including Kir2.1 channels. However, further studies are needed to clarify the important regulatory role exerted by nitrite at cardiovascular level.

In conclusion, our data have shown that SNO is a mechanism for nitrite to directly regulate cardiac proteins linked to various biological pathways and this is of particular importance in light of the emerging cardiovascular therapeutic role of nitrite. Indeed, there are several and remarkable advantages for using sodium nitrite over authentic NO gas or NO donor agents to treat cardiovascular diseases. Sodium nitrite is a highly stable compound and can be administered via inhalation, intravenous injection, intraperitoneal injection, and orally with NO only being released following the bioconversion of nitrite to NO in the circulation or ischaemic/hypoxic tissues. Despite the overwhelming enthusiasm for the clinical development of sodium nitrite for the treatment of cardiovascular disease, it is very important to proceed with caution as nitrite is not only an NO reserve but a bioactive agent directly involved in several physiological and pathological processes. The coexistence of two systems in physiological production of NO (NOSs and nitrite conversion) suggests that the cytoprotective and regulatory actions of nitrite are not limited to ischemic events but that nitrite plays a critical function distinct from the NOS-derived-NO and acts through different mechanisms, even under physiological conditions.
